# Enhancement and Compatibilization of Waste-Sourced Biocomposites Through Elastomer Blending and Matrix Grafting Modification

**DOI:** 10.3390/molecules29204905

**Published:** 2024-10-16

**Authors:** Shunmin Yi, Wanyu Liu, Shihua Xu, Ruijia Hu, Qing Li, Meijia Wu, Qingwen Wang, Zhimin Huang

**Affiliations:** 1 Guangxi Key Laboratory of Advanced Microwave Manufacturing Technology, Guangxi Academy of Sciences, Nanning 530007, China; 20200040@gxmzu.edu.cn (S.Y.);; 2 Guangxi Key Laboratory of Advanced Structural Materials and Carbon Neutralization, Guangxi Colleges and Universities Key Laboratory of Environmental-Friendly Materials and Ecological Restoration, School of Materials and Enviroment, Guangxi Minzu University, Nanning 530105, China; 3 School of Arts and Design, Wuyi University, Jiangmen 529020, China; 4 School of Materials Science and Engineering, Hubei University, Wuhan 430062, China; 5 College of Materials and Energy, South China Agricultural University, Guangzhou 510642, China

**Keywords:** biocomposites, grafting modification, interface adhesion, elastomer, deformation mechanism

## Abstract

A novel elastomer-modified multicomponent, multiphase waste-sourced biocomposites, was prepared for converting waste biomass and plastic into value-added products. The effects of blending elastomer–olefin block copolymer (OBC) and maleic anhydride (MAH), and divinylbenzene (DVB) co-grafting of recycled polypropylene (rPP) matrix on the adhesion interface, structure, and properties of high wood flour-filled (60 wt.%) composites were thoroughly investigated. The results indicated that DVB introduced branched structures into the polymer matrix molecular chain and increased the MAH grafting rate. Co-grafting rPP/OBC blends enhanced the interfacial adhesion among rPP, OBC, and wood flour. Additionally, MAH-grafted OBC was prone to encapsulating rigid wood flour, thereby forming an embedded structure. Notably, the tensile modulus and impact strength of the final three-component composites increased by 60% and 125%, respectively, compared with the unmodified composites. Additionally, dynamic mechanical analysis revealed that DVB-induced branching promoted the formation of microvoids in the OBC shell layer surrounding the wood, which in turn induced significant plastic deformation in the polymer matrix. This work offers a facile and efficient method for preparing high-toughness, high-stiffness, and low-cost waste PP-based composites for automotive interiors, and indoor and outdoor decoration.

## 1. Introduction

Biocomposites have recently experienced significant advancements in both academia and industry due to their favorable specific mechanical properties and eco-friendly characteristics [1,2]. Biocomposites can efficiently utilize natural lignocellulosic resources and plastic waste as important raw materials to produce high-value and sustainable products [3]. However, recycled plastic wastes, such as polypropylene (PP), polyethylene (PE), and polystyrene (PS), exhibit poor miscibility, and fully separating the individual components is technically difficult and uneconomic [4]. Additionally, most recycled plastic waste is a hydrophobic mixture and is incompatible with hydrophilic lignocellulosic particles [5]. The mechanical performance of the resulting waste plastic-based biocomposites generally fails to meet the performance requirements in structural and other engineering fields, particularly regarding impact strength, due to interphase incompatibility [6,7,8].

To better meet performance requirements and expand applications in emerging fields, various strategies for enhancing interfacial adhesion in biocomposites containing plastic waste have been extensively researched [9,10,11]. Coupling agents are an effective means to improve interfacial adhesion and mechanical properties in biocomposites [12]. However, the partial enhancement of the interface layer still falls short of the impact strength requirements for structural applications. Low impact strength, particularly at higher lignocellulose loadings (above 50 wt.%), remains a major drawback of biocomposites [13]. Consequently, this severely reduces the end-of-life performance of biocomposites. Incorporating strong hybrid fibers into biocomposites is an alternative strategy to enhance both stiffness and impact strength [14]. However, some drawbacks exist, such as poor interfacial compatibility between components and severe abrasion of processing equipment. Furthermore, reinforcing fibers, like carbon fiber, glass fiber, and Kevlar fiber, are not recyclable [15,16,17].

Blending modification with polyolefin elastomers or their maleated derivatives to improve impact strength has been a traditional, yet active, area of research, particularly for polyolefin-based biocomposites [18,19,20]. Extensive efforts devoted to elastomer-toughened polymer/filler composites have focused on achieving excellent toughness with minimal strength and modulus loss or even improvement [21,22,23]. In these multicomponent systems, two types of structures may form: elastomer and filler either separately distributed within the polymer matrix or the elastomer encapsulates the filler, forming an embedded structure [24,25]. Sudar et al. [26] chose two compatibilizers, maleated polypropylene (MAPP) and maleated ethylene–propylene–diene elastomer (MAEPDM), to prepare wood plastic composites separately. They found that only MAPP led to separate distribution of the components, whereas embedding was promoted by the introduction of MAEPDM. Molnar et al. [27] conducted a systematic study on composites with embedded structures, investigating their effects on stiffness and toughness. They reported that the stiffness of these composites is determined by the degree of embedding, while toughness increases can result from either an embedded structure or separate distribution. The toughness of these multicomponent systems is primarily determined by the dominant micromechanical deformation processes. Therefore, proper structural design is essential for developing high-performance materials [28,29,30]. Additionally, optimizing the composition, component type and content, and molding technology is a potential strategy to achieve greater stiffness and toughness in the final composites.

The structure–property relationships of PP/wood flour/elastomer composites prepared using a twin-screw extruder were reported in a previous study [31]. Polymer matrix modification with polar monomers can be used to control the structure of multicomponent composites. In addition to wood particle size and content, the properties of the interface layer impact the composite’s overall performance [32]. Moreover, the branched chains induced by the multifunctional monomer also influence the properties of the composites [33]. Although grafting virgin PP or PP/elastomer blends with maleic anhydride (MAH) resulted in satisfactory toughness and stiffness, the impact of these modifications on the structure and properties of recycled PP-based composites requires further investigation. The aim of our study was to determine the synergistic effect of elastomer blending and branched chain introduction on the impact resistance and stiffness of hybrid recycled PP/wood/elastomer composites. The structure of the three-component composites was controlled through matrix modification with MAH and the polyfunctional monomer divinylbenzene (DVB). The deformation and failure mechanisms of these composites were studied in relation to structure-related phenomena, such as the interface layer, branched chains, and embedding structure.

## 2. Results and Discussion

### 2.1. Study on the Structure and Properties of Modified Polymer Matrix

**Figure 1:**
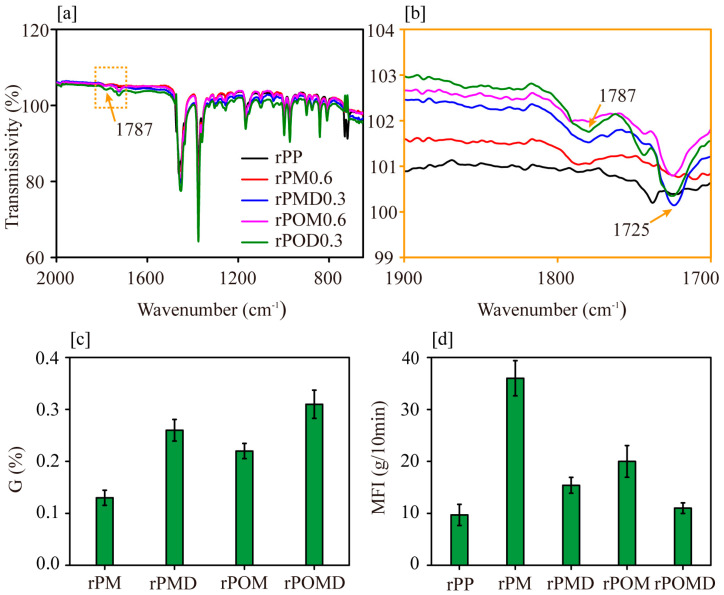
FTIR spectra (**a**,**b**), MAH grafting degree (**c**), and melt flow rate (**d**) of the modified rPP.

After melt grafting modification, new absorption peaks at 1787 cm^−1^ appeared for rPP (Figure 1a). These peaks corresponded to the carbonyl groups of cyclic anhydrides and the carboxylic acid formed after hydrolysis of the anhydride, indicating successful grafting of MAH onto the rPP molecular chain [34]. After the addition of DVB and OBC during MAH grafting, the carbonyl absorption peaks showed an increase at 1725 cm^−1^ (Figure 1b). This suggested that more MAH monomers were grafted onto the modified matrix. The increased MAH grafting rate aligned with the rise in carbonyl absorption intensity in the infrared spectra, confirming successful grafting of MAH onto the matrix molecular chain. After MAH/DVB co-grafting modification, rPP/OBC blends showed a higher MAH grafting rate than rPOM. (Figure 1c). The melt mass flow rate (MFI) of rPM increased compared with that of unmodified rPP (Figure 1d). This increase was attributed to the severe chain scission of rPP macromolecular chains caused by the initiator, which reduced the flow resistance of the rPM melt. The addition of DVB monomer and elastomer OBC inhibited rPP chain degradation, resulting in lower MFI values for rPMD and rPOM, although these values remained higher than those of rPP. Meanwhile, the MAH grafting rate increased. This suggests that the highly reactive DVB introduced branching in the matrix molecular chains, promoting MAH grafting [33,35]. Additionally, the polyolefin segments in the OBC chains facilitated MAH grafting and induced limited branching of macromolecular chains.

**Figure 2:**
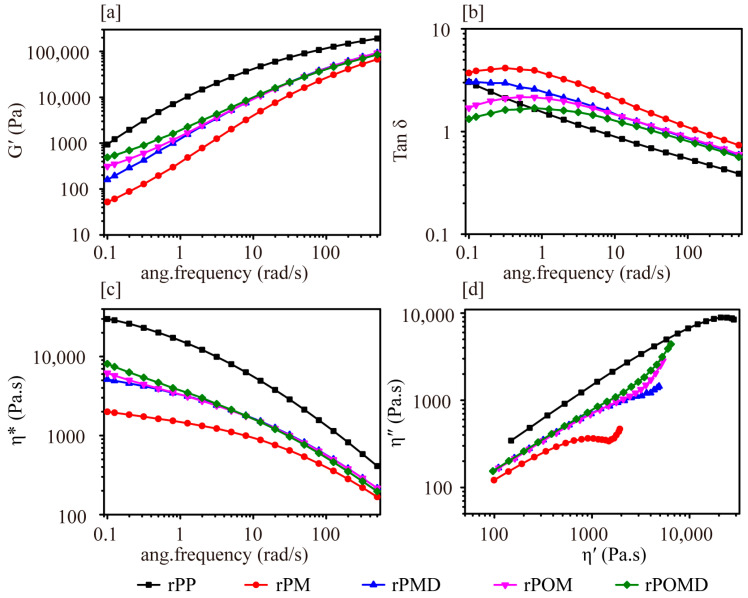
Rheological behavior of polymer matrix: (**a**) storage modulus; (**b**) loss damping; (**c**) complex viscosity; (**d**) loss viscosity–storage viscosity curves.

Compared with unmodified rPM, the storage modulus (G′) of rPOM and rPMD melts gradually increased in the low-frequency region, with a reduced slope at the curve’s end, more prominently in rPMD (Figure 2a). This increase was attributed to DVB-induced chain extension, which introduced branched structures into the rPP molecular chains [36]. Moreover, the additional incorporation of OBC promoted DVB-induced branching, enhancing chain entanglement and reducing the melt’s viscous deformation. In the low-frequency region, DVB addition reduced the tanδ of rPMD compared with rPM, making it lower than that of unmodified rPP. (Figure 2b). This suggests that DVB altered the topology of the rPP molecular chains, resulting in the formation of branched structures [35]. The branched structure with a strong chain entanglement effect prolonged the alpha relaxation of the rPP chain ends and gradually increased the elastic portion of the melt in the low-frequency region [37]. Following the additional incorporation of OBC, the tanδ of rPOMD in the low-frequency region was lower than that of rPMD. This suggested that OBC increased molecular chain entanglement in the melt, further enhancing the melt’s elastic response. As shown in Figure 2c, the chain scission process reduced the complex viscosity (η*) of rPM compared with that of unmodified rPP, which displayed distinct Newtonian fluid properties in the low-frequency region. The addition of DVB and OBC effectively inhibited the chain scission of rPP molecular chains, resulting in increased η* for rPMD and rPOMD. The presence of branched structures increased resistance to molecular chain flow orientation, enhancing melt shear thinning and exhibiting typical non-Newtonian fluid properties.

The straight-line portion of rPM in the Cole–Cole curve is shorter than that of unmodified rPP, attributed to the faster onset of short-chain relaxation (Figure 2d). The addition of DVB and OBC increased the lengths of the straight-line portions of rPMD and rPOMD in the Cole–Cole curve compared with rPM. This indicated that enhanced chain entanglement prolonged the relaxation time of the melt’s molecular chains. Additionally, the end region of the Cole–Cole curve began to warp upward, producing a trailing phenomenon, which signified a new relaxation process in the melt, distinct from that of linear polymers, demonstrating that DVB introduced branched structures into the rPP molecular chain [36]. The trailing phenomenon at the end of the Cole–Cole curve for rPOMD was more pronounced than that for rPMD, indicating that OBC caused DVB to introduce additional branched structures and some cross-linking to the matrix blends, leading to longer relaxation times for the molecular chains.

**Figure 3:**
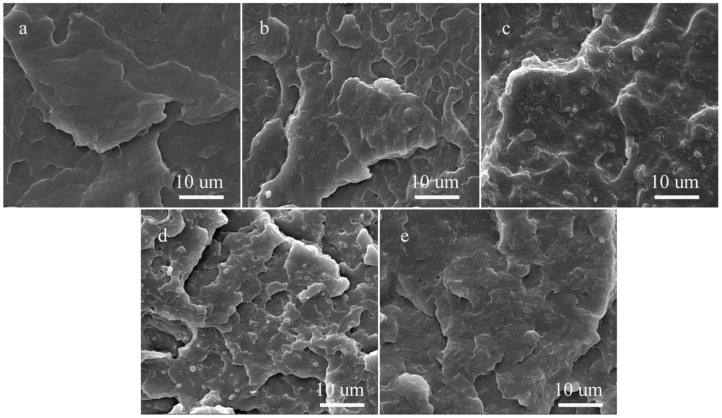
Fracture morphology of polymer matrix: (**a**) rPP; (**b**) rPM; (**c**) rPMD; (**d**) rPOM; (**e**) rPOMD.

After MAH graft modification alone, the rPM section exhibited numerous small blocky fracture layers and more crack bands than unmodified rPP (Figure 3), indicating typical brittle fracture. The addition of DVB monomer reduced the crack bands left after rPMD fracture compared with rPM (Figure 3c).

The morphology of these crack bands resembled viscous tear bands, which favored the ductile fracture of the polymer matrix. Following the addition of OBC, the rPOM fracture surface showed more brittle damage from short-chain PP, with crack bands present. The granular OBC particles and the matrix phase at the fracture surface primarily exhibited a “sea-island” structure [33]. The MAH grafting modification enhanced the interface compatibility between components, resulting in reduced debonding phenomena (Figure 3d). With the additional incorporation of DVB (Figure 3e), voids left by the debonding of OBC particles at the cross-section were reduced, and granular OBC particles nearly vanished. This suggests that MAH/DVB co-grafting modification enhances interfacial compatibility between PP and OBC.

**Figure 4:**
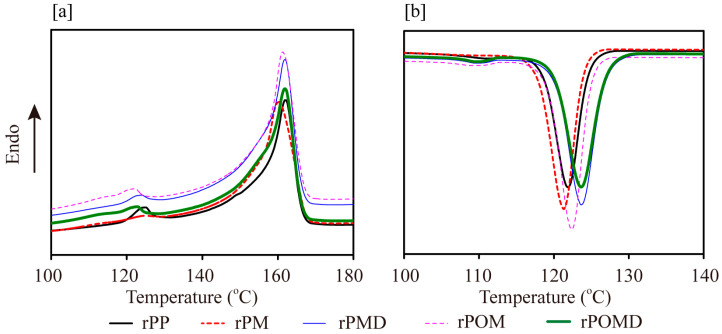
DSC curves of the polymer matrix: (**a**) melting; (**b**) crystallization.

Figure 4a shows that MAH graft modification caused degradation of the macromolecular chain, resulting in a shift in the melt peak of rPM toward lower temperatures. After the addition of OBC and DVB, the melting peaks of rPOM, rPMD, and rPOMD shifted to higher temperatures, though they remained lower than that of rPP. This shift could be attributed to the chain entanglement caused by the branched structures, which restricted the movement of rPP molecular chains at elevated temperatures [35]. However, the short chains generated during the degradation reaction still enhance molecular chain mobility at higher temperatures [36]. The crystallization curves in Figure 4b further showed that the MAH group, branched structure, and heterogeneous nucleation effect of OBC promoted the crystallization of rPP chain segments at higher temperatures.

Compared with unmodified rPP, the crystallization temperature (*T*_c_) of rPM decreased, while the crystallinity (*X*_c_) increased (Table 1). This was attributed to the difficulty of forming stable nuclei from short chains in rPM at high temperatures, while MAH heterogeneous nucleation accelerated crystal growth, resulting in increased X_c_ for rPM. With the further addition of DVB, melt temperature (*T*_m_), *T*_c_, and *X*_c_ of rPMD increased and reached their maximum compared with rPM. This could be attributed to the accelerated nucleation and crystal growth rates of rPP chain segments due to MAH and branched structures [36]. In rPP/OBC blends, although the presence of branched structures increased the *T*_c_ of rPOMD compared with rPOM, *X*_c_ still decreased. This decrease could be attributed to improved interfacial compatibility between PP and OBC, as well as chain entanglement between the molecular chains of the two components, which inhibited the movement of rPP chain segments. This inhibition blocked the ordered arrangement of rPP chain segments in the nuclei, reducing the growth rate of crystallite.

**Figure 5:**
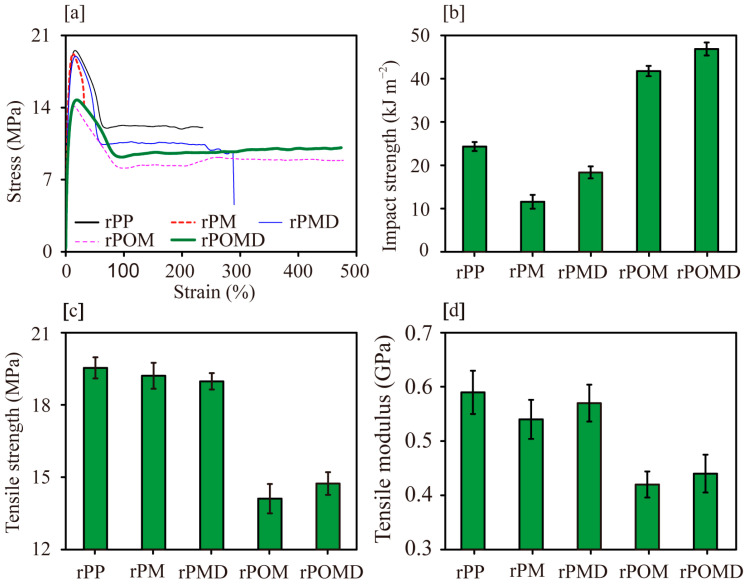
Mechanical properties of polymer matrix: (**a**) tensile stress–strain curves; (**b**) impact strength; (**c**) tensile strength; (**d**) tensile modulus.

The degradation of rPM during MAH melt graft modification resulted in reduced tensile properties and impact strength (Figure 5a). The further addition of DVB increased the tensile elongation at break, tensile properties, and impact strength of rPMD. This indicated that DVB inhibited macromolecular chain degradation and introduced branched structures into the matrix, thereby enhancing chain entanglement [37]. The enhanced intermolecular forces reduced slippage of molecular chains and improved resistance to external damage.

After adding OBC, the tensile elongation at break and the area below the stress–strain curves of rPOM and rPOMD increased compared with those of rPM, indicating an improvement in toughness. The material yielded under maximum tensile displacement of the universal mechanical testing machine without fracturing, indicating a significant improvement in toughness. This improvement could be attributed to the cavitation phenomenon of OBCs under external forces, which triggered significant shear yielding in the rPP matrix, thereby resulting in greater impact energy absorption and substantially higher impact strength compared with unmodified rPP [38]. However, the lower modulus of OBC led to reduced tensile modulus of rPOM compared with that of rPM. The further addition of DVB enhanced chain entanglement from the branched structure, resulting in increased impact strength, tensile strength, and modulus of rPOMD [36].

### 2.2. Properties of Composites

#### 2.2.1. Morphological Analysis

**Figure 6:**
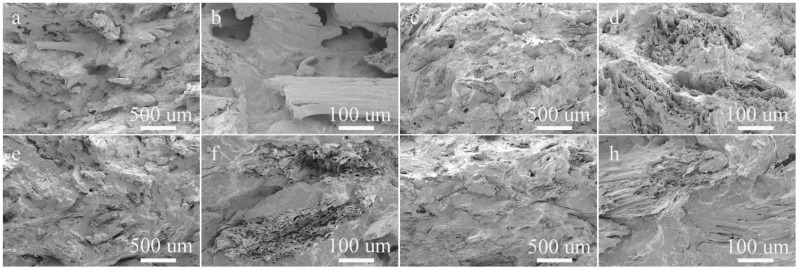
Fracture morphology of the composites: (**a**,**b**) WrP; (**c**,**d**) WrPM; (**e**,**f**) WrPOM; (**g**,**h**) WrPOMD.

The morphology of both unmodified and modified composites is shown in Figure 6. The section of WrP displayed numerous wood flour particles following debonding (Figure 6a). This revealed that the wood flour particles remained undamaged after debonding, and exhibited relatively smooth surfaces with no residual plastic matrix phase (Figure 6b). Additionally, a clear interfacial gap between the wood flour surface and the two phases of the matrix was visible in the WrP section. This indicated that the interfacial compatibility between wood flour and unmodified PP was extremely poor, resulting in an incompatible system [10]. The voids in the sections of the MAH graft-modified composites had largely disappeared (Figure 6). The interfacial gap between the wood flour and matrix in the graft-modified composites had disappeared, and the debonding of wood flour had decreased. Additionally, some wood flour particles remained embedded in the plastic matrix after damage [27] Additionally, the interfacial bond between the wood flour and the matrix appeared blurred, with no obvious interfacial gap observed. These observations indicated that the graft modification enhanced interfacial compatibility between the wood flour and the matrix components.

#### 2.2.2. Analysis of Mechanical Properties and Fracture Mechanism

**Figure 7:**
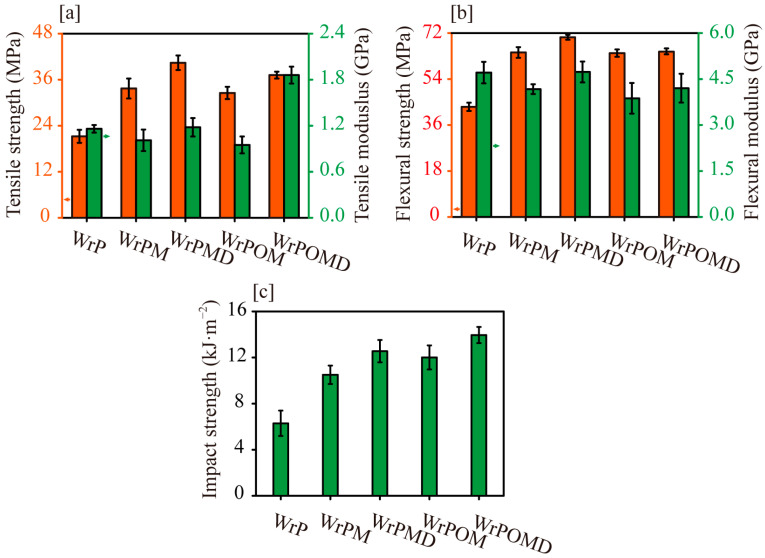
Mechanical properties of the composites: (**a**) tensile strength and modulus; (**b**) flexural strength and modulus; (**c**) impact strength.

The mechanical properties of the modified waste polypropylene/wood flour composites are presented in Figure 7. The tensile strength of WrPM increased by 59% compared with that of WrP, while the tensile modulus decreased by 13%. This increase was attributed to improved interfacial bond strength between the matrix and wood flour [39]. MAH graft modification improved the fluidity and polarity of rPM compared with unmodified PP, facilitating the dispersion of wood flour in the matrix and resulting in more extensive interfacial bonding within the composite. Under tensile stress, the interfacial layer facilitated stress transfer from the degraded PP short chains to the rigid wood flour [33]. Improved interfacial compatibility restricted short-chain molecular slippage at the interface, leading to more wood flour fracture and enhanced tensile strength of the composites during deformation. However, the lower modulus of rPM negatively impacted the composites’ resistance to deformation, leading to a tensile modulus lower than that of WrP. With the addition of DVB at the same MAH level, co-grafting further increased the tensile strength of WrPMD by 20% compared with that of WrPM, while the tensile modulus increased to match that of WrP. This improvement could be attributed to the increased grafted MAH groups on the matrix molecular chain, the introduction of branched structures, an expanded interfacial bonding area, and enhanced matrix strength, ultimately resulting in improved composite mechanical strength.

In the elastomer toughening system, the tensile strength and modulus of WrPOM and WrPM were nearly identical. The tensile modulus was shown to depend mainly on the content of the embedded structure formed by MAH-grafted elastomer-coated wood flours [26]. Therefore, the rigid embedded structure formed by MAH-grafted OBC-coated wood flour can partially mitigate the negative impact of the elastomer on the modulus [31]. In the DVB and MAH co-grafted blend matrix, WrPOMD showed a 10% increase in tensile strength and an 84% increase in tensile modulus compared with WrPM, and a 60% increase in tensile modulus compared with unmodified WrP. Co-grafting modification enhanced the interfacial compatibility among PP, OBC, and wood flour, improving stress transfer efficiency and thereby increasing the contribution of rigid wood flour to the composite’s mechanical properties, while offsetting some negative impacts of the low-modulus OBC [26,31]. Additionally, the extensive embedded structure increased the reinforcement efficiency of wood flour and enhanced the tensile modulus of the final composite material [25].

The flexural strengths of WrPM and WrPMD increased by 49% and 60%, respectively, compared with that of WrP (Figure 7b). This increase could be attributed to enhanced interfacial compatibility between wood flour and modified rPP, as well as improved matrix strength. For WrPM, despite the improved interfacial compatibility, the flexural modulus decreased by 11% compared with that of WrP. However, the addition of DVB restored the flexural modulus of WrPMD to match that of WrP. After adding OBC, the flexural strength and modulus of WrPOM were similar to those of WrPM. Further addition of DVB resulted in a smaller increase in the flexural strength of WrPOMD.

After MAH graft modification, the impact strength of composite WrPM increased by 69% compared with that of unmodified WrP. The impact strength of WrPMD was further improved by 20% with the addition of DVB co-graft modification (Figure 7c). This increase could be attributed to the enhanced grafting of MAH on the PP molecular chain, which reacted with the hydroxyl groups on the wood flour’s surface to generate more chemical bonds. Thus, the enhanced interfacial bond strength facilitated the material’s absorption of impact energy [39]. In addition, the effective interfacial compatibility and the entanglement of the branched structural chains inhibited the generation and expansion of cracks in the short chains under impact force [33]. The slippage of ungrafted short chains at the interface and within the matrix released the binding of the matrix molecular chains at high strain rates, promoting localized plastic yielding around them [31]. This enhanced the material’s energy absorption and increased the toughness of the co-graft-modified wood–plastic WrPMD. With the addition of OBC, the impact strength of WrPOM increased by 14% compared with that of WrPM. This improvement was attributed to microcracks triggered by cavitation at the OBC and the interface under external force, which promoted the shear yielding of the surrounding matrix [33,38]. Therefore, the absorption of a large amount of impact energy led to an increase in the impact strength of the final composites. Furthermore, the formation of embedded structures effectively enhanced the plastic yielding of the matrix and also improved the material’s capacity to absorb impact energy [27]. The addition of DVB further enhanced the impact strength of WrPOMD by 16% compared with that of WrPOM. The impact strength of WrPOMD increased by 33% and 11% compared with those of WrPM and WrPMD, respectively. This renewed increase in impact strength, combined with the increase in tensile modulus, could be attributed to the formation of an embedded structure [25]. The embedded structure reduced the ligament thickness of the matrix between the rigid wood flour particles and increased the effective elastomeric area, thereby promoting more shear yielding in the matrix, allowing the composites to absorb greater impact energy before damage occurs [26].

#### 2.2.3. Analysis of Dynamic Mechanical and Deformation Mechanism

**Figure 8:**
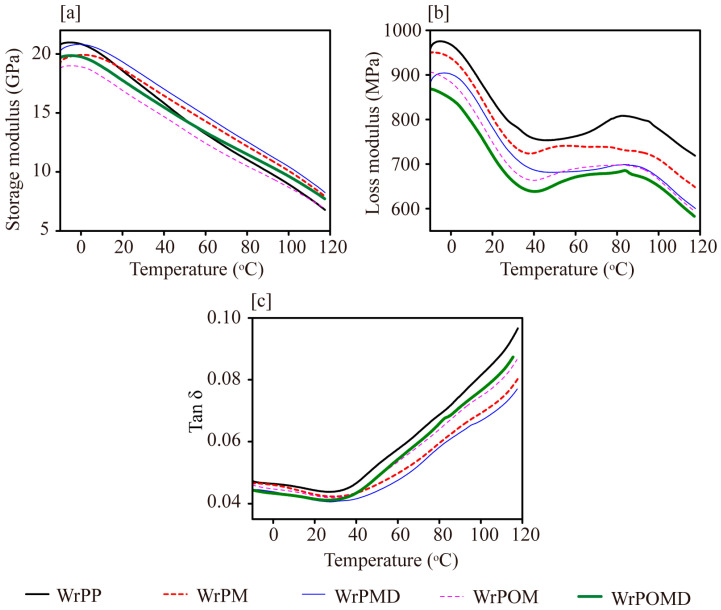
(**a**) Storage modulus, (**b**) loss modulus, and (**c**) loss factor of the composites.

As shown in Figure 8a, in the unmodified system, the G′ of unmodified WrP was consistently the highest across the tested temperature range. This was attributed to the molecular chain segments being in a “frozen” state at low temperatures, while the G′ of the composites was mainly contributed by the stiffness of the reinforced matrix and wood flour [22]. As the temperature increased, the kinematic activity of the matrix molecular chain segments increased, and weak interfacial bonding caused a rapid decrease in the G′ of WrP. Its value fell below those of WrPMD and WrPM at approximately 3 °C and 20 °C, respectively. In the WrPMD system, good interfacial compatibility and the branched structure of the matrix molecular chain showed a positive contribution to the composite’s modulus, resulting in a maximized G′ at high temperatures. Following the addition of OBC, its lower modulus resulted in the G′ value of WrPOM being the lowest, consistent with the trend observed in flexural modulus changes. The additional incorporation of DVB enhanced matrix strength and interfacial compatibility, partially offsetting the negative impact of OBC on modulus [33]. As a result, the G′ of WrPOMD exceeded that of WrP at elevated temperatures.

The magnitude of the loss modulus G″ primarily reflected energy dissipation from the movement of molecular chains at the composite interface (Figure 8b). As the temperature increased, energy dissipation from matrix molecular chain relaxation increased the G″ of the composites. The loss modulus showed two transition peaks around 0 °C and 80 °C. The relaxation peak at 0 °C was primarily associated with the polymer’s glass transition. The differences in the glass transition relaxation peaks at low temperatures were small. This was attributed to interface interactions, which restricted chain segment movement. The relaxation peak at 80 °C corresponded to molecular chain movement in the crystalline region of rPP. With increasing temperature, rPP molecular chain mobility improved after activation. In WrP, weak interfacial bonding increased rPP chain relaxation, while friction with wood flour in the interface increased G″ due to increased energy dissipation. After MAH grafting modification, chain movement restriction reduced at higher temperatures, thereby enhancing the short-chain mobility in rPM. This facilitated movement in the crystalline matrix, shifting the onset and peak relaxation to lower temperatures. In addition, after DVB co-grafting modification, both onset and peak relaxation of the composites shifted to higher temperatures compared with WrPM. After MAH grafting modification of the rPP matrix, the G″ of both WrPM and WrPOM decreased. This indicated that improved interfacial compatibility effectively restricted molecular chain movement at the interface, reducing energy loss. In the MAH/DVB co-grafted system, as the number of MAH groups in the matrix molecular linkages increased and branched structures formed, the movement of molecular chains at the interface became more difficult, leading to a decrease in G″ for WrPMD and WrPOMD.

The tanδ of unmodified WrP was consistently the largest (Figure 8c). This phenomenon could be attributed to the poor dispersion of wood flour and weak interfacial bonding between the components, resulting in significant energy dissipation within the weak interfacial layers and defects in the composites [40]. The co-grafting of modified rPP with MAH and DVB improved interfacial compatibility and reduced defects, resulting in lower tanδ values for the composites WrPM and WrPMD. The further addition of OBC increased the tanδ values of both WrPOM and WrPOMD in the high-temperature region, while the tanδ value of WrPOMD was only lower than that of WrP. This increase could be attributed to the presence of a strong interfacial layer, which promoted cavitation within the OBC and the formation of voids at the interface as the temperature rose, leading to enhanced energy dissipation from the matrix shear yielding process [31]. After the addition of DVB, the chain entanglement from the branched structure inhibited the slip of short chains, further enhancing the shear yielding process triggered by the OBCs, as evidenced by the increased tanδ value. In addition, the branched structure in the matrix could effectively promote local plastic deformation of the elastomer interface layer around wood flour, which consumed much more energy. This massive plastic deformation occurring around the embedded structure would finally enhance the impact strength of the composites. This result was consistent with the static mechanical property findings.

## 3. Experimental

### 3.1. Materials

The post-consumer recycled polypropylene random copolymer (rPP) used in this study was sourced from Dongguan Haiyi Plastic Raw Materials Co., Ltd. (Dongguan, China). The olefin block copolymer (OBC, 9010) was supplied by Dow Chemical Company (Midland, MI, USA), with a melt flow rate (MFR) of 0.5 g/10 min at 230 °C under a 2.16 kg load. The initiator (dicumyl peroxide, DCP), monomers (MAH, DVB), solvents, and lubricant were reagent-grade products supplied by Tianjin Bochen Company (Tianjin, China). Wood flour (Populus alba, 30–100 mesh) was prepared in the laboratory using a specialized crusher.

### 3.2. Grafting of Polymer Matrix

Melt-grafting reactions were performed using an AK53 co-rotating twin-screw extruder (Nanjing Rubber and Plastics Machinery Co., Ltd., Nanjing, China) with an L/D ratio of 56. The polymer matrix mixed with monomers (MAH, DVB) and initiator (DCP) in a specific mass ratio was fed into the extruder (Table 2). The temperatures in the melting section (zones 1–4) were set to 140 °C, 160 °C, 170 °C, and 180 °C, respectively. The reaction section (zones 5–10) was maintained at 190 °C, and the vacuum section (zones 11–12) was set to 200 °C. The mold temperatures in zones 13 and 14 were set to 180 °C and 170 °C, respectively. The main motor speed was set to 20 Hz, and the feeding speed was 5.7 kg/min, with a vacuum level of 0.1 MPa in the barrel.

### 3.3. Preparation of Waste-Sourced Biocomposites Sample

Wood flour (WF) was dried in a vacuum oven at 103 °C for 24 h. The polymer matrix (virgin or grafted) and WF were homogenized at the mass ratio specified in Table 3 using an SJH30 twin-screw extruder (Nanjing Rubber-Plastic Machine Ltd., Nanjing, China) with an L/D ratio of 36. The zone temperatures were set to 150 °C, 160 °C, 170 °C, 180 °C, 180 °C, 170 °C, and 160 °C. The granulated materials were crushed and used to prepare composite plates via a single-screw extruder (L/D ratio of 8, SJ45, Nanjing Rubber-Plastic Machine Ltd., Nanjing, China). The plates were 40 mm wide and 4 mm thick. The zone temperatures were set to 150 °C, 160 °C, 170 °C, 175 °C, 180 °C, and 178 °C.

### 3.4. Characterization and Testing

The purified maleic anhydride-grafted polypropylene was prepared by dissolving the modified polypropylene in xylene at a concentration of 1 g/100 mL. The solution was heated and refluxed at 140 °C for 1.5 h, and an excess of acetone was added to precipitate the polymer, followed by rinsing. The maleic anhydride grafting rate (G) was determined via rapid titration of the purified sample in hot xylene, using phenolphthalein as an indicator and a potassium hydroxide-ethanol standard solution [41]. The purified polymer sample was hot-pressed into a film at 190 °C and analyzed using attenuated total reflectance (ATR) on a Nicolet 6700 Transform Infrared Spectrometer (Nicolet Company, Madison, WI, USA).

The melt mass flow index (MFI) was measured using an XRZ-400 tester (Jilin University Teaching Instrument Factory, Changchun, China) under the conditions specified in GB3682-2000 [42] (at 230 °C and 2.16 kg). The shear rheological properties of the polymer samples before and after modification were tested using a Discovery HR-2 rotational rheometer (TA Instruments Inc., New Castle, DE, USA), with the temperature set to 185 °C. The final gap was 2 mm, strain amplitude was 1%, and the angular frequency scanning range was 0.1 rad/s to 500 rad/s. The melting and crystallization behaviors of the polymer matrix (5 mg) were analyzed by differential scanning calorimetry (DSC, Diamond DSC, Perkin Elmer, Shelton, CT, USA) using a heating/cooling rate of 10 °C/min in the temperature range of 60–200 °C under nitrogen flow. The crystallinity of rPP samples was calculated using the following equations [33]:

$$X_{c}=\frac{∆H_{f}}{∆H_{f}^{0}}\times 100\%$$

where $∆H_{f}$ is the melting enthalpy value of the samples and $∆H_{f}^{0}$ is the melting enthalpy of the hypothetically 100% crystalline rPP [37].

Tensile and flexural tests were conducted using a universal mechanical testing machine (RGT-20A, ARI Medical Technology Co., Ltd., Hong Kong, China) to assess the mechanical properties. In accordance with ASTM D 638-10 [43], the plastic tensile specimens were dumbbell-shaped, with dimensions of 165 × 13 × 4 mm^3^ and a tensile rate of 50 mm/min. The wood–plastic sheet was prepared into dumbbell-shaped specimens (165 × 13 × 4 mm^3^) for testing, with a tensile rate of 5 mm/min and a gauge length of 50 mm. The flexural specimens were rectangular (80 × 13 × 4 mm^3^) according to ASTM D790-10 [44], with a crosshead speed of 2 mm/min and a span of 64 mm. Notched impact specimens of the plastic matrix (80 × 13 × 4 mm^3^, notch depth 2.7 mm) were tested using a Charpy impact tester (XJ-50G, Chengde Precision Testing Machine Co., Ltd., Chengde, China) following ASTM D256-10 [45]. The pendulum energy used was 5 J. Impact specimens of the composites (80 × 10 × 4 mm^3^) were tested with a span of 60 mm and a pendulum energy of 2 J using a Charpy impact tester (XJ-50G, Chengde, China) according to ASTM D4812-06 [46]. For each of the tests mentioned, eight specimens were tested. A dynamic mechanical analyzer (Q800, TA Instruments Inc., New Castle, DE, USA) was used to investigate the dynamic viscoelastic behavior of the composite. Specimens (35 × 13 × 4 mm^3^) were tested in the single-cantilever mode with a 50 μm amplitude, 1 Hz frequency, and a temperature range from −10 °C to 120 °C at a heating rate of 3 °C/min.

A scanning electron microscope (Quanta 200, FEI Company, Hillsboro, OR, USA) was used to observe the morphology of modified polypropylene. The samples were frozen in liquid nitrogen for 5 min and immediately broken by the fixture, and further sputtering with a thin gold layer at an acceleration voltage of 15 kV.

## 4. Conclusions

This work demonstrated that the modification method combining OBC blending and MAH/DVB co-grafting could be used to prepare highly wood flour-filled waste polypropylene-based composites with excellent toughness and rigidity. In the MAH grafting modification system, the addition of DVB not only inhibited PP degradation, but also promoted MAH grafting and introduces branched structures. Therefore, in the composites, grafting modification of the matrix enhanced interfacial compatibility among rPP, OBC, and wood flour, promoting the formation of embedded structures. The extent of embedding of wood into maleated elastomer–OBC was proven by the higher tensile modulus of the composites. The synergistic effect of the effective interfacial layer and the branched structure enhanced the shear yielding process of the OBC-initiated matrix. Compared with the unmodified composites, the tensile strength, flexural strength, impact strength, and tensile modulus of the modified ternary hybrid composites increased by 75%, 39%, 125%, and 60%, respectively, after elastomer blending and MAH/DVB co-grafting modification. This approach is significant for recycling waste plastics and enhancing the application value of wood–plastic composites.

## Figures and Tables

**Table 1:** Crystallization data of polymer matrix.

Samples	*X*_c_ (%)	*T*_m_ (°C)	*T*_c_ (°C)
rPP	37.1	162	122.0
rPM	45.2	160.1	121.4
rPMD	46.4	161.8	123.8
rPOM	42.6	161.3	122.4
rPOMD	42.0	161.8	123.7

**Table 2:** Formulation of the polymer matrix.

Samples	rPP (wt.%)	OBC (wt.%)	MAH ^※^ (wt.%)	DVB ^※^ (wt.%)	DCP ^※^ (wt.%)
rPP	100	0	0	0	0
rPM	100	0	0.6	0	0.09
rPMD	100	0	0.6	0.3	0.09
rPOM	90	10	0.6	0	0.09
rPOMD	90	10	0.6	0.3	0.09

^※^: Percentages of DCP, MAH, and DVB in the total mass of the polymer matrix.

**Table 3:** Formulation of the composites.

Sample	WF (wt.%)	Polymer Matrix (wt.%)	Lubricant ^※^ (wt.%)
WrP	60	rPP(40)	2
WrPM	60	rPM(40)	2
WrPMD	60	rPMD(40)	2
WrPOM	60	rPOM(40)	2
WrPOMD	60	rPOMD (40)	2

^※^: PE wax lubricant accounts for 2% of the total mass of the matrix and wood flour.

## Data Availability

Available data are presented in the manuscript.
